# Three-dimensional motion analysis of the thumb after trapeziometacarpal joint surgery

**DOI:** 10.1177/17531934251393568

**Published:** 2025-11-20

**Authors:** Sophie Charlotte Brackertz, Gabriella Fischer, Maurizio Calcagni, Lisa Reissner

**Affiliations:** 1 Division of Plastic Surgery and Hand Surgery, University Hospital Zurich, Zurich, Switzerland; 2 Institute for Biomechanics, ETH Zurich, Zurich, Switzerland; 3 Division of Hand Surgery, Balgrist University Hospital, University of Zurich, Zurich, Switzerland

**Keywords:** 3D motion analysis, LRTI, TMC joint, Touch dual mobility prosthesis, trapeziectomy, trapeziometacarpal joint surgery, trapeziometacarpal osteoarthritis, Wilson osteotomy

## Abstract

**Introduction:**

The purpose of the study was to describe thumb kinematics and function during basic motion tasks and activities of daily living following different surgical techniques for trapeziometacarpal (TMC) joint osteoarthritis using a three-dimensional motion capture system.

**Methods:**

Included were 24 patients (29 thumbs) following Wilson osteotomy, Touch prosthesis arthroplasty and trapeziectomy with ligament reconstruction and tendon interposition (LRTI) at a minimum of 12 months postoperatively.

**Results:**

During basic motion tasks, it was observed that patients after LRTI showed alterations in most of the assessed movements, while osteotomy and Touch patients showed a preserved range of motion. Altered mobility of the TMC joint was more evident in the combined movements of opposition or circumduction compared with standard anatomical planes. In activities of daily living, we found a similar range of motion of the trapeziometacarpal joint in all groups, but different movements in the adjacent joints, including marked hyperextension of the metacarpophalangeal joint in the LRTI group.

**Conclusion:**

Our study found different preservation of the maximal ROM following different TMC joint procedures. Information about the higher preserved range of motion following osteotomy and arthroplasty may be additional factors for consideration when choosing a procedure. Three-dimensional assessment of thumb kinematics after various trapeziometacarpal joint surgeries can enhance our understanding of expected outcomes and can also aid in postoperative assessment and patient education.

**Level of evidence:**

III

## Introduction

Trapeziometacarpal joint osteoarthritis (TMC OA) is a common degenerative condition, and its implicated restricted motion and pain may result in a notable loss of function (Eaton and Littler, 1973; Teunissen et al., 2022). Surgical treatment options for TMC OA range from first metacarpal osteotomy and arthroplasty with joint prosthesis to trapeziectomy with or without ligament reconstruction and/or tendon interposition (Cootjans et al., 2017; Hamasaki et al., 2021; Kadiyala et al., 1996; Vermeulen et al., 2011). However, not all techniques can be equally offered to the same patient groups owing to different stages of OA.

Moreover, there is sparse information on the impact of different surgical techniques for TMC OA on biomechanics, and widely used patient-reported outcomes do not indicate the expected range of motion (ROM) and impact on activities of daily living (ADL). Motion remains difficult to measure for the TMC joint, and while the Kapandji score might be best, its precision remains low (Jha et al., 2016). Furthermore, TMC joint function is not only defined by motion but also by strength and should not only be assessed by standard ROM but also by ADL. Today, the 3D motion-capture system is the most accurate way of dynamic ROM assessment, and it has been increasingly applied to measure motion of the hand (Fischer et al., 2020; Metcalf et al., 2008; Reissner et al., 2019a; Tanaka et al., 2024). Still, the kinematics of the thumb during complex tasks combined with grip strength is understudied, and we do not know what function can be restored following TMC joint surgery.

A reliable treatment for early TMC OA is the Wilson metacarpal closing wedge osteotomy. This procedure shifts the load from the volar aspect of the joint towards the healthier dorsal surface (Pellegrini et al., 1996) and has been shown to provide good clinical outcomes (Chou et al., 2014; Kriechling et al., 2022; Nüesch et al., 2024; Parker et al., 2008; Tomaino, 2000). Good clinical results were also noted after TMC joint implant arthroplasty (Cootjans et al., 2017; Falkner et al., 2023; Herren et al., 2023b; Lussiez et al., 2021). Trapeziectomy with ligament reconstruction tendon interposition (LRTI) was long considered the reference standard treatment for TMC OA (Kadiyala et al., 1996; Vermeulen et al., 2011). Patients achieved a reliable outcome but often without regaining strength (Ulrich-Vinther et al., 2008; Vadstrup et al., 2009).

Hence, with numerous techniques, we need better information to explain to patients what to expect from surgery in different stages of OA with different techniques. The aim of this study was to describe the postoperative biomechanics and function of the thumb after different surgical interventions for TMC OA. We decided to perform 3D motion analysis and test ADL under load after three techniques.

## Methods

### Participants and protocol

Thumb kinematics of 24 patients (18 women, 6 men) with a median age of 53 years (30–60 years) were assessed using a 3D motion capture system. Patients who underwent TMC joint surgery for osteoarthritis with Wilson osteotomy, Touch prosthesis or LRTI were recruited from three different hand surgery departments. Minimum time after surgery was 12 months assuming that the ROM had stabilized by that time. Patients experiencing bilateral thumb OA who had both TMC joints treated were eligible for inclusion. The cohort comprised joint motion of 29 thumbs (23 women, 6 men). Only patients aged between 18 and 60 years were eligible for study inclusion. Preoperative TMC OA stages were classified and documented using the Eaton–Littler classification (Eaton and Littler, 1973). Selection of the surgical technique was based on the OA stage and the department practice. The study was approved by the local ethics committee, and all participants provided written consent for their data to be used for this analysis (Kek-ZH-Nr: 2015-0395).

### Surgical technique

All operations were performed by expert level 3–5 surgeons (Tang and Giddins, 2016). For the implant arthroplasty, all patients received a dual mobility prosthesis (Touch^®^, Kerimedical SA, Plan-les-Ouates, Switzerland). Only 15° angulated stems were implanted. For the osteotomy, 1.5 LCP condylar plates (DePuy Synthes, Oberdorf, Switzerland) were inserted. For LRTI, one of two techniques (Epping and Noack (1983) or modified Weilby (1988)) was used, depending on the surgeon’s preference.

### Setup and experimental protocol

**Figure 1.**
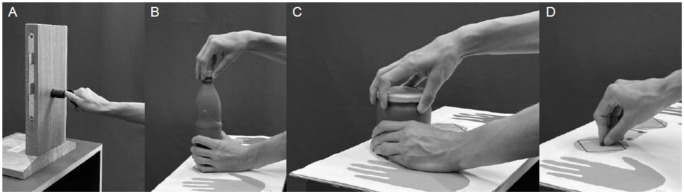
Illustration of movements during assessed activities of daily living: (a) key turning; (b) opening and closing a bottle; (c) opening and closing a jar; and (d) grasping a small object.

An optoelectronic motion capture system consisting of 20 fixated infrared cameras (Vicon^®^ Vero v2.2 motion capture system, Oxford Metrics Ltd, UK, with a resolution of 2048 × 1088 pixels) and the corresponding software Vicon Nexus (version 2.12) was used for data collection. To assess the motion of all hand and finger joints simultaneously, 47 reflective markers with diameters of between 3 and 9 mm were placed on specific positions on the finger, wrist, and forearm as previously described by Fischer et al. (2020) and Reissner et al. (2019b). Recordings were carried out with a frequency of 100 Hz. Initially, a static reference position was captured, whereby the hand was lying with a 40°-angled wedge in the first web space to define neutral joint position (Fischer et al., 2020). Afterwards, the following basic motion tasks (BMTs), aiming to measure maximal ROM of the thumb joints, were performed: (a) flexion/extension (palmar/radial abduction); (b) adduction/abduction; (c) opposition/retropulsion; and (d) circumduction. For flexion/extension, the start was a neutral position, and then the thumb was fully flexed across the palm and then fully extended. For adduction/abduction, the hand was placed flat on the table, and the thumb was radially abducted and adducted three successive times. For circumduction, three circular movements with the thumb at a maximal range were performed, and opposition was carried out analogous to Kapandji. Furthermore, a set of ADL (Figure 1) was performed to measure thumb kinematics during functional movements: (a) key turning; (b) opening and closing a bottle; (c) opening and closing a jar; and (d) grasping a small object. Each participant performed five trials of each BMT and ADL task.

**Figure 2.**
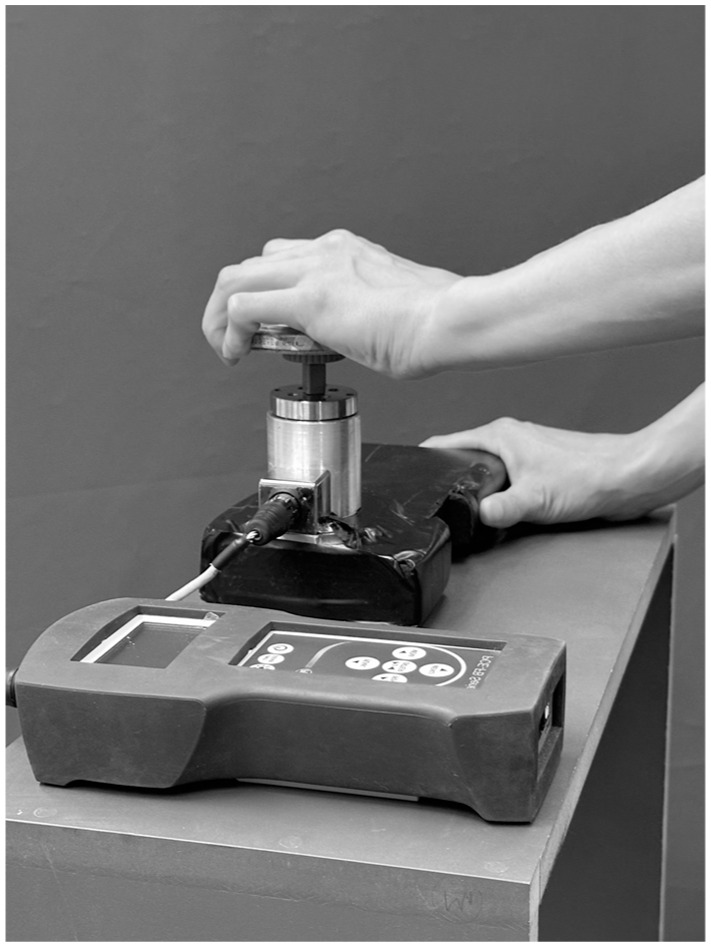
Torque device to measure peak torque during different activities of daily living.

In addition to 3D motion analysis, strength was measured by a torque device measured in Newton metres (Figure 2) (Reissner et al., 2021). Strength during ADL was also recorded for the contralateral side in order to allow reporting of grip strength of the patient’s affected hand as a percentage of the contralateral side. Hence, we report torque during ADL only for those patients having assumingly healthy contralateral TMC joints. In the osteotomy group, five patients had bilateral surgery for TMC OA; in the Touch and LRTI groups, this affected four patients each.

### Kinematic model and data analysis

The kinematic model of the hand we used was previously published (Reissner et al., 2019b) and is based on validated measurements of healthy control subjects. The post-processing and labelling of the recorded data were done with Vicon Nexus. Further data analysis was done using MATLAB (R2022b, MathWorks, USA). No data filtering was applied. The kinematic assessment was based on marker clusters. This methodology was previously described in detail by Fischer et al. (2020). Using the segmental approach, joint rotations were described as the relative rotation matrix of the distal segment with respect to the proximal segment. As distinguishing between movements of the TMC and the scaphotrapeziotrapezoidal joint is not possible with skin markers, we refer to the movements of the first metacarpal. However, we assumed that the main movement takes place in the TMC joint, because it has the greatest mobility.

The ROM for the TMC joint, metacarpophalangeal (MCP) joint and the interphalangeal (IP) joint during BMTs are reported for the three operated groups. The ADL were subdivided into three phases: reaching the object, turning/holding phase and returning to the starting position. Time points were determined manually in the 3D recordings. Whereas BMTs are reported using the ROM, for ADL we were interested in the joint angle used to grasp the different objects. Hence, the grip position during the ADL was defined as the joint angle in the middle of the holding/turning phase (mean of 20 frames). Time normalization was performed for visual comparison of the joint kinematics.

We decided on a primarily descriptive approach, since the three surgical groups are inherently different and do not allow for a comparison. Finally, the relation between the maximal MCP joint extension during the BMT and the (hyper-)extension during the ADL and the relation between MCP joint extension in BMTs compared with ADL and torque measurements were assessed.

## Results

### Participants/demographics

We included a total of 24 patients (18 women, 6 men) with a median age of 53 years (30–60 years). Five patients had bilateral thumb OA, hence 29 affected thumbs were included. The median age of patients with osteotomy and Touch prosthesis was 6 years younger compared to the LRTI patients. Defined by the surgical technique, patients with osteotomy had less severe OA (Table 1).

**Table 1.** Patient characteristics.

	Wilson osteotomy (*n* = 10)	Touch prosthesis (*n* = 9)	LRTI (*n* = 10)
Sex (female)^a^	8	7	8
Age (years)^b^	52 [30-57]	52 [45-56]	58 [49-60]
Eaton stage of OA^a^			
I	4	-	-
II	6	5	5
III	-	4	4
IV	-	-	1
Follow-up (months)^b^	30 [12-57]	18 [13-31]	33 [12-71]

a Data are presented as n. ^b^Data are presented as median [range]. LRTI = Ligament reconstruction and tendon interposition.

### Maximal range of motion of the thumb joints

**Figure 3.**
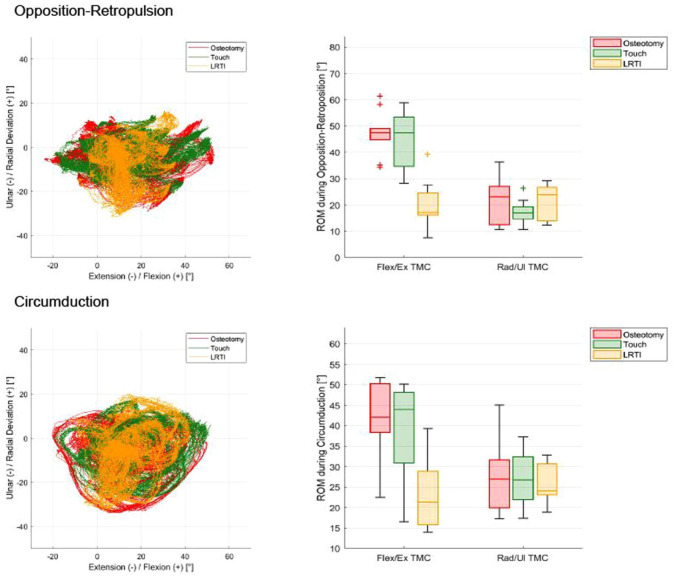
Combined basic motion tasks for the trapeziometacarpal joint. Left: motion in extension/flexion plotted against the ulnar and radial deviation plane. Right: distribution of data for each group in boxplots.

The ROM for the TMC, MCP and IP joints during BMTs is reported in Table 2. For the combined motion of opposition–retropulsion and circumduction, the ROM of the TMC joint for the different groups is shown in Figure 3. The ratio between TMC flexion–extension and radial–ulnar deviation ROM during thumb opposition was 5:2 for the osteotomy and Touch group, while it was 1:1 in the LRTI patients.

**Table 2.** Thumb range of motion during dynamic flexion–extension and adduction–abduction task.

Joint	DOF	Wilson osteotomy (*n* = 10)	Touch prosthesis (*n* = 9)	LRTI (*n* = 10)
IP	Flexion–extension	86 [55–98]	80 [76–95]	83 [64–97]
	Flexion	54 [29–64]	48 [28–68]	48 [40–51]
	Extension	39 [28–44]	32 [28–50]	41 [24–47]
MCP1	Flexion–extension	71 [62–85]	70 [58–78]	71 [64–78]
	Flexion	66 [57–74]	54 [47–62]	52 [43–63]
	Extension	6 [−4–15]	9 [3–17]	19 [10–26]
TMC	Flexion–extension	44 [37–50]	39 [30–49]	31 [27–32]
	Flexion	31 [28–39]	33 [22–42]	25 [15–31]
	Extension	10 [6–17]	6 [4–9]	2 [−2–13]
TMC	Radial–ulnar	18 [13–22]	17 [15–20]	14 [13–20]
	Radial deviation	10 [8–14]	9 [8–11]	9 [6–11]
	Ulnar deviation	6 [6–10]	7 [6–9]	6 [4–8]

Data are presented as median degrees [interquartile range]. DOF = Degrees of freedom; IP = interphalangeal; LRTI = ligament reconstruction and tendon interposition; MCP = metacarpophalangeal; TMC = trapeziometacarpal.

### Kinematics of the thumb during ADL

**Figure 4.**
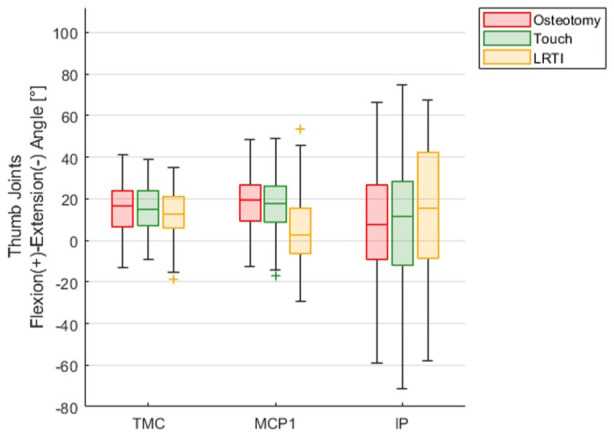
Joint grip angles of thumb joints over all activities of daily living.

The grip positions for the thumb TMC, MCP and IP joints are summarized for all four ADL and are displayed in Figure 4. Overall, the TMC joint showed a similar baseline motion in all ADL and between groups. However, MCP joint flexion during the ADL differed for the LRTI group. The IP joint, independent of the treatment group, showed a high variability between patients.

When looking at the different ADL separately, we found the TMC joint flexion angle during execution of the turning/gripping movements ranged between 0° when opening/closing a bottle with maximal strength and 24° when picking up a small object using pinch grip. This corresponds to 0–78% of the maximal flexion of the joint determined during the BMT. Only five LRTI patients had a TMC flexion in the BMT less than 24°. Still, two osteotomies, one Touch prosthesis and four LRTI patients almost reached their individual maximally possible TMC joint flexion (less than 5° to the end position of the joint), mainly during pinch grip. In addition, 19 patients even exceeded their individual maximum TMC joint extension of the BMT during the ADL, in particular when turning the lid of a jar with force. When turning a key, the TMC joint showed similar kinematics in all three groups. We found this to be true for the unloaded and loaded conditions. Whereas the osteotomy and Touch groups showed similar motion in the MCP joint, the LRTI patients showed an extended to partially hyperextended MCP joint. When applying maximum force this hyperextension became even more pronounced for the LRTI group. In opening a bottle, the LRTI group again showed a marked hyperextension of the MCP joint. This was most pronounced in opening with force. Two subjects from the Touch group showed considerably increased flexion of the MCP when opening a bottle with maximal force. We found no differences in the kinematics of the TMC joint when opening and closing a jar. Considering only patients with MCP joint extension greater than 20° (same limit as in Schindele et al., 2025), all of the LRTI (6/6) and osteotomy (2/2) patients were also affected from MCP joint hyperextension in the ADL’s, while two Touch patients showed no hyperextension when performing ADL.

### Grip strength

Strength measured in percent of the contralateral side during the ADL is shown in Table 3. Only 16 patients were available for comparison of grip strength during ADL, as five patients had bilateral thumb OA and three were partially missing force measurements.

**Table 3.** Forces ADL/key pinch.

	Wilson osteotomy (*n* = 5)	Touch prosthesis (*n* = 5)	LRTI (*n* = 6)
Opening jar^a^	113 [89–138]	95 [88–109]	78 [63–91]
Closing jar^a^	93 [75–138]	93 [88–98]	86 [76–105]
Opening bottle^a^	93 [46–96]	79 [71–86]	59 [51–94]
Closing bottle^a^	80 [64–97]	69 [59–112]	65 [47–144]
Turn key CCW^a^	93 [72–108]	106 [89–125]	72 [57–86]
Turn key CW^a^	94 [93–98]	113 [99–123]	54 [50–85]
Key pinch			
vs. contralateral	94 [93–110]	113 [96–156]	76 [63–86]
absolute^b^	7.0 [6–8]	6.8 [5–8]	4.1 [4–6]

Data of torque strength during ADL/key pinch vs. contralateral are presented as median percentage of contralateral side [interquartile range].

a Data of torque during ADL were measured in Nm.

b Data of key pinch absolute forces are presented as kg [interquartile range].

ADL = Activities of daily living; CCW = counterclockwise, close; CW=clockwise, open; LRTI = ligament reconstruction and tendon interposition; Nm = Newton metre.

## Discussion

This study uses 3D motion analysis and grip strength during functional tasks to allow for a description of thumb kinematics after different surgical techniques for TMC OA. The maximal ROM in BMTs in the osteotomy group was high, while patients after LRTI had less TMC mobility. Altered mobility was more evident in the combined movements of opposition or circumduction compared with the standard anatomical planes. In ADL, contrary to what we expected, the groups showed similar flexion angles in the TMC joint but different MCP joint motion patterns.

Our patients, similar to other study cohorts, were predominantly women but in a markedly lower median age compared to most other studies (Herren et al., 2023a; Hoogendam et al., 2023; Lussiez et al., 2021). Patients undergoing osteotomy showed lower preoperative Eaton–Littler OA stages I–II, and the only patient with Eaton–Littler stage IV was treated with LRTI (II–IV). Most patients (16/29) had preoperative stage II.

When assessing the ROM in the standard TMC joint flexion–extension plane, the LRTI group was restricted to a median of 31°, which is slightly higher than previously published data from a 3D motion analysis by Tanaka et al. (2024). In thumb circumduction, where one can see the whole arc of movement of the thumb, LRTI patients also showed a severely restricted TMC flexion–extension. The osteotomy and Touch arthroplasty patients performed similarly during circumduction. The osteotomy patients showed almost no alteration in thumb opposition. We found the opposition movement in the osteotomy and Touch patients to consist of a markedly larger proportion of flexion combined with a smaller radial deviation movement of the TMC joint. In contrast, LRTI patients had more restricted TMC joint flexion. The LRTI patients had a notable change in the motion pattern, with the MCP thumb joint also being affected. Whether the altered motion coupling in the MCP joint is related to the LRTI technique or was present before surgery needs to be assessed in future studies.

Looking at all ADL, the required TMC joint flexion usually remained below 80% of the maximal flexion determined in the BMT. Hence, the required TMC flexion during the ADL rested within the capability of most patients. Picking up a small object in the pinch grip was the most challenging task regarding TMC joint flexion, whereas opening/closing a jar with maximal strength required the most TMC joint extension. Trapeziometacarpal joint extension during ADL often exceeded the extension during the BMT. Exerting torque against an object led to a passive extension in the TMC joint while the active TMC joint extension was measured during the BMT.

Finally, a tendency for a hyperextension deformity of the MCP joint in the LRTI group was observed, while the osteotomy and Touch group showed no significant MCP joint compensatory motion. Across the ADL, the MCP joint hyperextension in the LRTI group was most pronounced when turning a key and highest when using force. However, different activities showed a variance in hyperextension mechanisms that were not clearly limited to a single ADL or use of force. We found that the larger the central object in the ADL (jar > bottle > key), the greater the difference in hyperextension of the MCP joint. A secondary hyperextension deformity of the MCP joint as previously described in patients after trapeziectomy by Robles-Molina et al. (2017). In contrast, Falkner et al. (2023) and Schindele et al. (2025) recently published a marked normalization of the preoperative hyperextended MCP joint in their 55 and 37 implanted Touch prostheses, possibly owing to the restoration of the thumb length. Our data align with these observations, even though preoperative values were not available to evaluate the true effect of the chosen procedure. Degeorge et al. (2018) found a dynamic MCP hyperextension instability during maximal pinch force execution to be increased in 11 patients with postoperative MCP joint hyperextension. However, it remains unclear whether MCP joint hyperextension in X-rays or ROM measurements are also associated with hyperextension during functional movements. This study revealed a strong relation between the maximal MCP joint extension during the BMT and the (hyper-)extension during the ADL for the LRTI group and a trend for the osteotomy patients, whereas the Touch patients showed MCP (hyper-)extension in BMT but not during the ADL. Further studies with more patients are needed to determine whether there is a relation with the normalization of preoperative hyperextension and to better understand the underlying mechanisms.

The present study had some limitations. The number of patient was limited but still bigger than in other 3D kinematic studies (Tanaka et al., 2024). These measurements were complex and time consuming, making it difficult to use them in larger cohorts. Another limitation is the missing preoperative measurements of the TMC joint and MCP joint ROM, making it difficult to quantify the changes of the different surgical techniques. Postoperative kinematics is most likely influenced by the preoperative condition. To what extent it is influenced by the surgical technique needs further investigation. Finally, some surgical details (e.g. the position of the cup in the trapezium or the scapho-metacarpal distance) might also play a major role in determining the final ROM. A future prospective study aims to address the aforementioned limitations by comparing 3D motion in ADL pre- and postoperatively and correlating data with radiologic findings.
